# Bengt Westermark and our current understanding of tumor pathogenesis

**DOI:** 10.3109/03009734.2012.670978

**Published:** 2012-04-19

**Authors:** Robert A. Weinberg

**Affiliations:** Whitehead Institute for Biomedical Research Ludwig/MIT Center for Molecular Oncology MIT Department of Biology CambridgeMA 02142USA

The field of cancer research has advanced so far over the past four decades—the precise time-span of Bengt Westermark's career—that it would be unrecognizable to those who last visited it in 1972. At that time, we knew that cancer cells grew uncontrollably and that a number of carcinogens were likely to be genotoxic mutagens, i.e. that among their other functions they worked to induce cancer through their ability to damage the genes within human tissues. That represented the entirety of our insights into the molecular and cellular mechanisms underlying cancer formation.

As it turned out, many of the insights in this area flowed from an unanticipated area. In the 1970s, enormous efforts were invested in trying to find a variety of viruses that served as the causal agents of human cancer. These viruses—so the thinking went—infected cells in human tissues, transformed them into cancer cells, and thereby triggered the outgrowth of tumors. The discovery of retrovirus reverse transcriptase in 1970 helped to feed this frenzy, including, in the United States, the 1971 launching of Richard Nixon's War on Cancer.

The flurry of activity searching for the retroviral agents causing human cancer ultimately failed, but it left a rich repository of information on genes that were associated with rapidly transforming retroviruses, and it was these genes, stolen from the genomes of normal cells, that ultimately opened up the field of molecular oncology, more precisely the research that revealed the molecular origins of human cancer.

These genes—proto-oncogenes and oncogenes—turned out to be central players in triggering the outgrowth of human cancers. Mutant cellular growth-promoting genes were found to be the central drivers forcing cancer cells to proliferate inexorably. Of special interest was the research that undertook to place these genes and their protein products in the context of normal cell physiology. The question therefore became: How do normal cell growth-promoting genes become subverted by the process of tumor pathogenesis?

It is precisely this question that focused the attention of Bengt Westermark, beginning 35 years ago. In a prescient paper, he revealed that platelets, which play a central role in blood-clotting and wound repair, release factors that stimulate the growth of normal glial cells. This implicated these factors in driving the proliferation of normal cells and, quite possibly, that of neoplastic cells.

By the late 1970s his group had led the charge to identify a discrete growth factor—platelet-derived growth factor (PDGF)—as a central agent in this stimulation and the presence of a distinct receptor on the surface of normal cells—the PDGF receptor—that enables a variety of normal cells to respond to PDGF in their surroundings. This work, driven forward by the powerful collaboration between Bengt Westermark and Carl-Henrik Heldin, provided the conceptual framework for understanding how the subversion of such a cellular growth-stimulating pathway could lead to the runaway proliferation of cancer cells. At the same time, it revealed why and how serum was such a critical ingredient in the complex media in which cells were cultured then and now.

By 1985, their work led to the realization that cancer cells often secrete growth factors, notably PDGF, which then function on the same cells that released them, inducing these cells to proliferate. This led to the further insight that certain oncogenes, notably the *sis* oncogene of a simian sarcoma virus, function in this *autocrine* manner to drive cell proliferation. (This simian sarcoma virus was another fruit of the 1970s stampede to find cancer-causing human retroviruses). Stated differently, unlike normal cells, which depend on growth factor stimulation by their neighbors before they undertake proliferation, cancer cells will often make their own growth-stimulatory (mitogenic) factors with which they alter their surrounding microenvironment and stimulate their own proliferation.

This theme of growth factor-mediated stimulation of neoplastic cell growth has threaded its way through Bengt Westermark's opus in the succeeding three decades and helps to explain why Uppsala became one of the world's leading centers in this type of research. To be sure, his research expanded to encompass other types of growth-stimulating factors, such as epidermal growth factor (EGF) and thyroid-stimulating hormone, and thus further extended and reinforced the earlier research of this powerful group. Because of the central role of PDGF signaling, this research forms the core of our current understanding of how gliomas—commonly occurring brain cancers—acquire the ability to grow relentlessly, with an invariably fatal outcome. The Westermark opus also provides many indications—specific molecular targets—of how one can develop novel therapeutic agents against brain tumors.

In my own case, I followed this research closely since the mid-1970s, because it was so important and so critical to forming my own conceptualization of how cancer begins. It is for this reason that I was especially honored and flattered to have been recognized by colleagues in Uppsala as the recipient of an honorary doctorate from an institution that has come to represent one of the world's leading centers in the area of molecular cancer research!

